# An ensemble approach integrating LSTM and ARIMA models for enhanced financial market predictions

**DOI:** 10.1098/rsos.240699

**Published:** 2024-09-11

**Authors:** Lesia Mochurad, Andrii Dereviannyi

**Affiliations:** ^1^ Department of Artificial Intelligence, Lviv Polytechnic National University, Kniazia Romana str., 5, Lviv 79905, Ukraine

**Keywords:** forecasting, financial markets, neural networks, machine learning, ensemble learning

## Abstract

Forecasting financial markets is a complex task that requires addressing various challenges, such as market complexity, data heterogeneity, the need for rapid response and constant changes in conditions, to gain a competitive advantage. To effectively address these challenges, it is necessary to constantly improve existing and develop new methods of intelligent forecasting, which will improve the accuracy of forecasts, reduce risks and increase the productivity of financial decision-making processes. In this article, we study and analyse forecasting methods in financial markets, such as support vector regression (SVR), autoregressive integrated moving average (ARIMA), long short-term memory recurrent neural network (LSTM) and extreme gradient boosting algorithm (XG-Boost). Based on this analysis, we propose an ensemble forecasting procedure that integrates LSTM and ARIMA models. Due to the careful combination of these models, our approach yields better results than individual methods. For example, our model demonstrates a significant 15% improvement in root mean square error (RMSE) and a slight improvement in coefficient of determination compared with LSTM. Furthermore, simulation results obtained on three real-world datasets and evaluated using the RMSE criterion confirm the superiority of our proposed method over alternative methods such as LSTMs, transformer models and optimized deep recurrent neural networks with long short-term memory for financial market forecasting. Furthermore, our approach creates the prerequisites for parallelizing both models, thus providing an opportunity to accelerate forecasting results in future research.

## 1. Introduction

The financial market is a set of exchange and redistribution processes of buying and selling financial resources for the implementation of production and financial activities. They form complex dynamic systems, constantly affected by various economic, political, social and technological factors in unpredictable ways, making it difficult to predict their dynamics [1].

Due to the nature of financial data, such as daily stock or index prices, they can be characterized as a time series of numerical values, where each value represents a price or other financial indicator at a specific point in time. There are many ways to predict time series of varying complexity, from the most primitive machine learning methods to sophisticated models and neural network systems [2].

The application of such methods for forecasting financial markets involves analysing historical data on prices, volumes and other indicators of financial instruments, such as stocks, currencies and bonds, and creating models to forecast the future dynamics based on these data [3,4]. These methods can be used both individually and in combination with other methods to obtain more accurate and reliable financial market forecasts.

Forecasting accuracy of financial markets is highly important. Investors and traders rely on it to make decisions about buying or selling financial assets and develop optimal investment strategies to avoid unexpected losses and ensure profitability. High forecasting accuracy contributes to the development of financial markets by ensuring more efficient allocation of capital and stimulating investment activity [5].

Accordingly, it can be concluded that financial markets can be characterized by numerical series in the context of analysing stock prices, exchange rates, trading volumes and other financial data [6]. Financial data, such as daily stock or index prices, are time series of numerical values, where each value represents a price or other financial indicator at a particular point in time.

**Figure 1.**
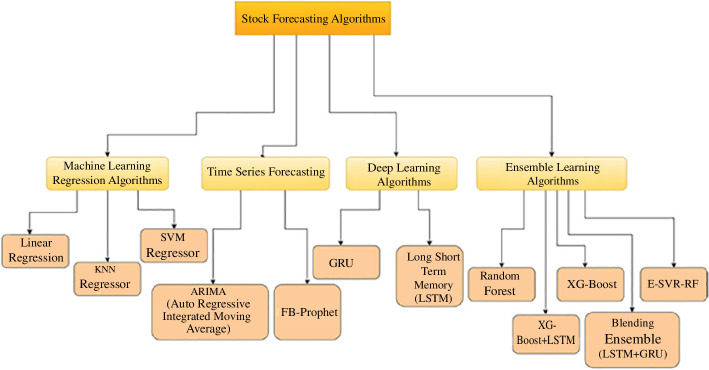
Financial market forecasting methods. E-SVR-RF, Ensemble Support Vector Regression - Random Fores; FB, Facebook Prophet; GRU, gated recurrent unit; KNN, K-Nearest Neighbors; SVM, support vector machine; XG-Boost, extreme gradient boosting.

Based on an analysis of scientific articles, the most popular approaches for financial market forecasting are machine learning regression algorithms, time series statistical models, deep learning and ensemble algorithms (see figure 1) [4,7–9].

Machine learning regression algorithms look for a relationship between a set of input data and a target variable—a value to be predicted. Linear regression is a classic representative of this approach. This algorithm tries to find the optimal linear relationship between the variables—in the form of a straight line. However, this algorithm is sensitive to outliers and ineffective when working with complex and dynamic datasets [10]. That is why it is usually used to identify general trends to quickly assess the superficial situation in the market [11].

Due to the potential complexity of the data and the unpredictability of financial markets, nonlinear regression algorithms are used alongside linear regression, for example, support vector machine regressor (SVR). The kernel functions of this algorithm seek the curve where the error between the predicted and true values is the smallest [12]. The ability to process both linear and nonlinear data makes this algorithm a useful tool for various real-world applications, including finance, economics, engineering and so on. SVR is less sensitive to over-training because it uses regularization, can work with high-dimensional and sparse data, and is less vulnerable to outliers in the data because it uses support vectors to define the regression function.

However, using support vector regression can be computationally intensive: the computational and memory requirements increase exponentially with the number of features and instances to be trained. Also, SVR performance is highly dependent on the choice of kernel function and its parameters, so significant cross-validation may be required to optimize it [13], and irregular or noisy data, the presence of missing values and multivariate collinearity make it difficult to apply it to dynamic tasks and real-time predictions [14].

Statistical models that use time series as input parameters fall into the category of univariate analysis. Such models include the autoregressive model with a moving average (ARMA) and its modification with an integrated moving average (ARIMA), which was developed for long-term forecasting. By applying differentiation operations, an ARIMA model can transform non-stationary data into stationary data, which can then be processed using autoregressive components and moving averages to predict possible market developments [15].

One significant limitation of using statistical models to analyse financial markets is that they are based on the assumption that the current patterns will continue in the future. However, this assumption is more likely to be true in the short term than in the long term, so statistical models provide fairly accurate forecasts for the near future but lose reliability as the forecast period increases. For the same reason, statistical models cannot be reliable at high rates of trend change and cannot predict turning points [16].

Deep learning algorithms are often used to predict financial market changes because of their ability to identify temporal dependencies and patterns in historical price data. These algorithms learn the dynamics of financial indicators from historical data and apply those patterns to the data at hand in order to predict future market behaviour. Deep learning can take into account not only linear but also nonlinear dependencies between factors that affect the financial market, as well as adapt to changing market conditions Most often, gated recurrent units (GRUs) and long short-term memory recurrent neural networks (LSTMs) are used for this purpose [17]. Both networks are designed to solve the problems associated with long-term dependence and the disappearance of gradients. At the same time, GRU has a simpler architecture and requires fewer parameters compared with LSTM, which allows it to better identify short-term dependencies and work with less data. This partially mitigates one of the main limitations of this approach: conventional recurrent neural networks require a large amount of data for effective training, and stock market data are usually limited, especially for longer-term predictions. Insufficient data can lead to over-fitting when the model fails to effectively generalize new data [18].

Still, deep learning algorithms are vulnerable to market shocks. As mentioned earlier, financial markets are driven by complex causal relationships, making them extremely volatile, with sudden and unpredictable price movements. GRUs and LSTMs rely on patterns learned from historical data, so they may have difficulty responding to rapid changes in market conditions. To overcome these limitations, additional techniques such as feature engineering or external data sources (news analysis) are often used. In addition, risk management and historical data-backtesting strategies are important to ensure the reliability and robustness of any stock market forecasting system [19].

Ensemble approaches to forecasting in financial markets combine multiple models to produce more accurate and reliable results. They improve accuracy by combining different approaches to data analysis, which allows for the discovery of complex relationships and improves model versatility. Ensemble methods also reduce the risk of over-fitting and increase the stability of predictions by using different modelling strategies.

They can be divided into two main types: bootstrap and boosting algorithms. Bootstrap algorithms, such as Random Forest, train several models on different subsets of the same data and then average their predictions. This reduces the variability of individual models and makes them less prone to over-training. At the same time, the extreme gradient boosting algorithm (XG-Boost) and other boosting algorithms train several models sequentially so that each new model tries to correct the errors of the previous ones, which improves adaptability [20]. Due to these features, ensemble learning algorithms can handle complex and nonlinear data, reduce the risk of choosing the wrong or suboptimal model for a particular problem, and improve the generalization and performance of the learning system. However, this approach is computationally expensive and time-consuming, as well as difficult to interpret.

No matter what approach is chosen, the big problem for creating accurate forecasting models is limited and inaccurate data. To successfully predict market behaviour, it is necessary to have access to sufficient quality and up-to-date data on the past and current market situation, as well as on the expectations and sentiments of market participants. However, some data sources may be unavailable, incomplete, outdated, contradictory or erroneous, which reduces the quality and accuracy of the forecast.

Another pitfall can be over-training and over-optimization of the models for the available data, there may be a problem with low model performance in real-world conditions. Such behaviour is caused by the over-optimization of model parameters when they are selected in a way that maximizes a certain metric on the validation data but does not take into account possible changes in market conditions, which results in a loss of generalization ability.

Nevertheless, due to the dynamism and dependence on external conditions characteristic of financial markets, a prediction may be wrong regardless of the model’s actions [21].

In today’s fast-moving financial environment, the ability to predict market trends not only accurately but also quickly is crucial. Traditional methods often fail to process the huge volumes and high velocity of financial data, resulting in delays and reduced accuracy [22]. To solve these problems, there is a need to create prerequisites for parallelization and acceleration of the forecasting process in general based on certain modern parallel or distributed computing technologies. This would allow simultaneous processing of large datasets and complex models, significantly reducing computing time and increasing the overall efficiency of forecasting systems.

The purpose of this paper is to investigate existing intelligent methods for forecasting financial markets and ways to improve them to ensure high forecasting accuracy.

The main results of this work are listed in the following paragraphs.

— Several methods for forecasting financial markets were analysed and applied, and using the two best ones, an ensemble method was developed for prediction.
— The effectiveness of the proposed method is compared with the state-of-the-art intelligent forecasting methods in financial markets based on three datasets, with clear quantitative indicators of the advantages of the former.
— An approach to parallelization of model training in an ensemble is developed, which will allow efficient use of parallel and distributed computing resources and significantly speed up the forecasting process.

The practical significance of the proposed approach lies in its applicability for developing automated robotic systems that interact with financial markets, enabling autonomous trading decisions based on market forecasts, thus leveraging methods like LSTM and ARIMA to adapt to market complexities, promptly respond to changes and gain competitive advantage through enhanced prediction accuracy, ultimately assisting in risk management and enhancing productivity amid the dynamic nature of financial markets.

Section 2 contains an overview and preprocessing of the input data, as well as a description of the proposed methodology, providing information on the methods used to create the ensemble learning approach. Section 3 demonstrates the results of numerical experiments and engages in discussion after applying the approach. Finally, §4 provides the main conclusions and suggestions for further research.

## 2. Materials and methods

### 2.1. Review and preprocessing of dataset

To test the effectiveness of the proposed forecasting algorithm, we chose the Huge Stock Market Dataset [23], which is based on complete historical daily price and volume data for all stocks traded on the NYSE, NASDAQ and NYSE MKT exchanges in the United States. You can choose from catalogues with data from exchange-traded investment funds, as well as shares of 7195 different companies for different periods.

Data preprocessing is an important step that precedes model training and includes cleaning, transformation and integration of raw data to make it suitable for analysis [24]. The purpose of data preprocessing is to improve its quality and adapt it to a specific task. The first step is to identify and remove trends from the data under study. This way, we can isolate cyclical and irregular data properties, isolate them from the overall trend, and identify obvious patterns and relationships in the data. This is an important step when working with time series, such as financial market data, because by removing the trend, we essentially remove the noise that can prevent us from making accurate predictions [25]. The exponential model was found to be a much better fit for the data used, so we will use it to remove the trend. But first, let us check for moving data using the augmented Dickey–Fuller test obtained for the obtained trend [26], the results of which are presented in table 1.

**Table 1.** Results of the trend study using the extended Dickey–Fuller test.

overall assessment		−3.02445
*p*‐value		0.032674
number of delays		36
the number of reviews required to calculate critical values		7583
critical values (%)	1	−3.43121
	5	−2.86192
	10	−2.56697

As can be seen in table 1, the overall score is −3.02445, and the *p*-value is 0.032674. The critical values for the 1%, 5% and 10% significance levels are −3.43121, −2.86192 and −2.56697, respectively, which means that there is still moving data in the time series because the overall estimate is not smaller than all the critical values. Many models and methods of time series analysis assume that the data are somewhat stationary, and therefore the mean and autocorrelation are constant, but the variance may vary.

**Figure 2.**
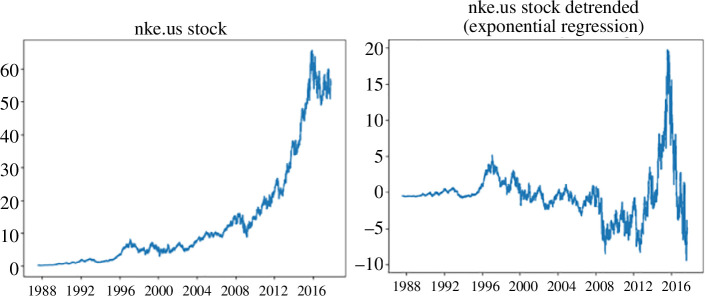
Graphical representation of Nike shares (nke.us) before and after removing the trend based on the exponential model.

To remove the trend from our data, we subtract values, predicted by the exponential model, from the actual data (see figure 2).

**Figure 3.**
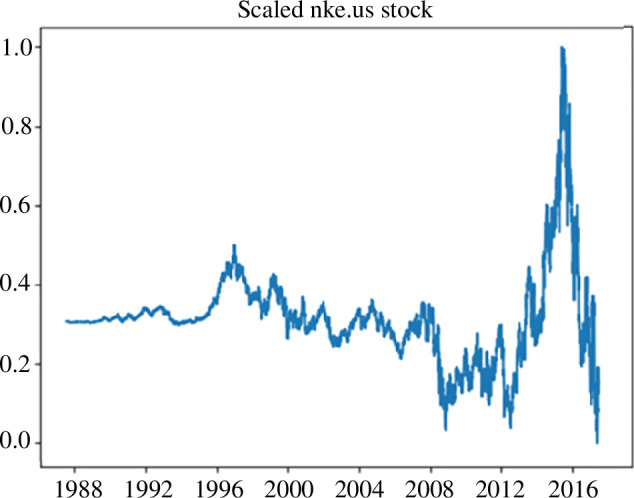
Graphical representation of the normalized values of Nike shares (nke.us) after trend removal.

The next step is to normalize the data. In this case, we use min–max scaling, which transforms the stock data to a range from 0 to 1. Thus, we preserve the original distribution of the data while transforming it to the specified range, and normalize the data for use in algorithms and neural networks [27]. Figure 3 shows the results of data normalization.

### 2.2. Description of the proposed ensemble forecasting procedure

The first stage of the proposed forecasting algorithm involves data loading and preprocessing. Missing values are replaced by the average of the neighbouring values, and then the inherent data is determined. Next, the coefficients of determination for the linear and exponential trend of the data are found to determine how to remove the trend. However, if no coefficient meets the threshold of 0.95, then the data does not have a clear linear or exponential trend, because it is, for example, too variable or even chaotic. In this case, the trend is removed by the first difference method, which consists of finding the difference between consecutive values in the time series [28]. This transformation creates a new series that represents the changes between each consecutive data point. In this way, we can partially remove the main trends of the dataset and introduce stationary data into such a numerical series.

The next step is to normalize the data. As described earlier, we use min–max scaling, which transforms the stock data to a range from 0 to 1. This preserves the original distribution of the data while transforming it to the specified range, and normalizes the data for use in algorithms and neural networks.

The data are divided into training, validation, and testing data in the proportion of 80/10/10. To do this, we use a time split because we need to preserve the sequence of values in the time series. This ensures that the temporal order during train–test splits is maintained and models do not try to identify underlying patterns from random disconnected samples. The data in each set are combined into number of groups 90 days each. The number of days depends on the data themselves and represents the period, during which the partial autocorrelation between the first and last values of the time series should have decreased to a sufficiently insignificant level so as not to harm the model. Thus, for Nike shares (nke.us), we settled upon a group of 90 days of data, which will be used to predict the value of the 91st day. After that, the group is shifted one day into the future and reformed, this time to predict day 92. This continues until the last actual value in the time series has a corresponding group of the previous 90 days. This way, we prevent any possible data leakage and increase the amount of data available for training. Although duplication of training data leads to over-fitting and worsens the generalization ability of the model, in our case, this risk is minimized, as division into groups and constant shift into the future allows algorithms to perceive values in constantly changing context, thus identifying new similar patterns rather than reinforcing those already learned.

Alternatively, we may use cross-validation when the amount of data is just too small to split it three-way into training, validation and testing sets. Although cross-validation is quite a basic approach, it allows us to avoid reducing the number of samples which can be used for learning the model, and therefore the results are less likely to depend on a particular random choice for the pair of sets. All because the training set is split into smaller sets called ‘folds’. A model is trained using *n* − 1 of these sets as training data and the resulting model is validated on the remaining part of the data by measuring the resulting accuracy. This approach can be computationally expensive but does not waste too much data, which is a major advantage when available financial data are limited.

Then, in the second stage, the data are submitted to the proposed forecasting method for processing. As a result of a preliminary analysis of existing state-of-the-art intelligence methods, including their performance, a recurrent neural network with long short-term memory and a statistical autoregressive model with a moving average were chosen as the basis of our method. These models work in a process similar to ensemble learning algorithms: each model receives training, validation and testing sets of data, separately and independently attempts to identify underlying patterns, dependencies and trends, validates training results and builds its prediction of the financial market, after which the results are generalized. By this, we mean finding the arithmetic mean of the results of both models, which will be the result of our proposed model.

**Figure 4.**
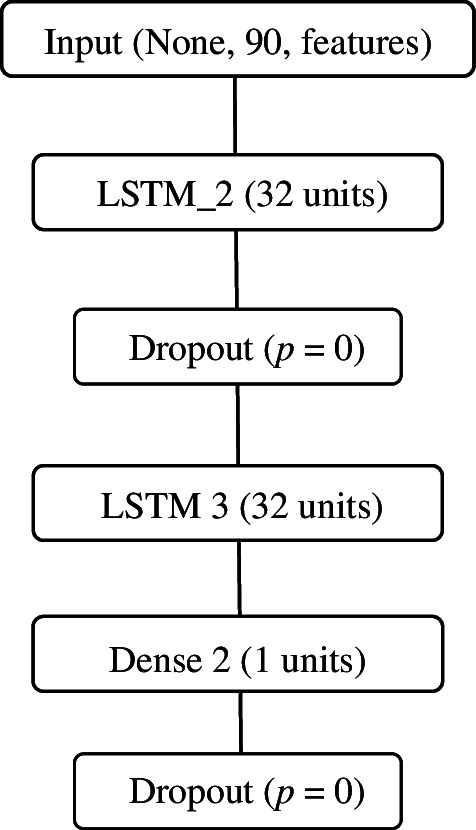
Structure of our own LSTM model.

Our recurrent neural network with long short-term memory consists of two LSTM layers, for 4352 and 8320 parameters, respectively, separated by a dropout layer. This results in a fairly lightweight recurrent neural network with a long short-term memory of only 12 705 training parameters, which makes the training data requirements quite low and is great for financial market data, which is usually limited, especially for longer-term predictions. A schematic representation of the proposed LSTM model is shown in figure 4.

In our model:

— The first LSTM layer (LSTM_2) has an input shape (none, 90, features) and an output shape (none, 90, 32). Number of parameters: 4352.
— The dropout layer has a dropout rate of 0, so the output size remains the same and the number of parameters is 0.
— The second LSTM layer (LSTM_3) has an input shape (none, 90, 32) and an output shape (none, 32). Number of parameters: 8320.
— The Dense layer (Dense_2) has an input size (none, 1). Number of parameters: 33.

In total, the number of parameters in our model is 12 705, all of which are trained.

**Figure 5.**
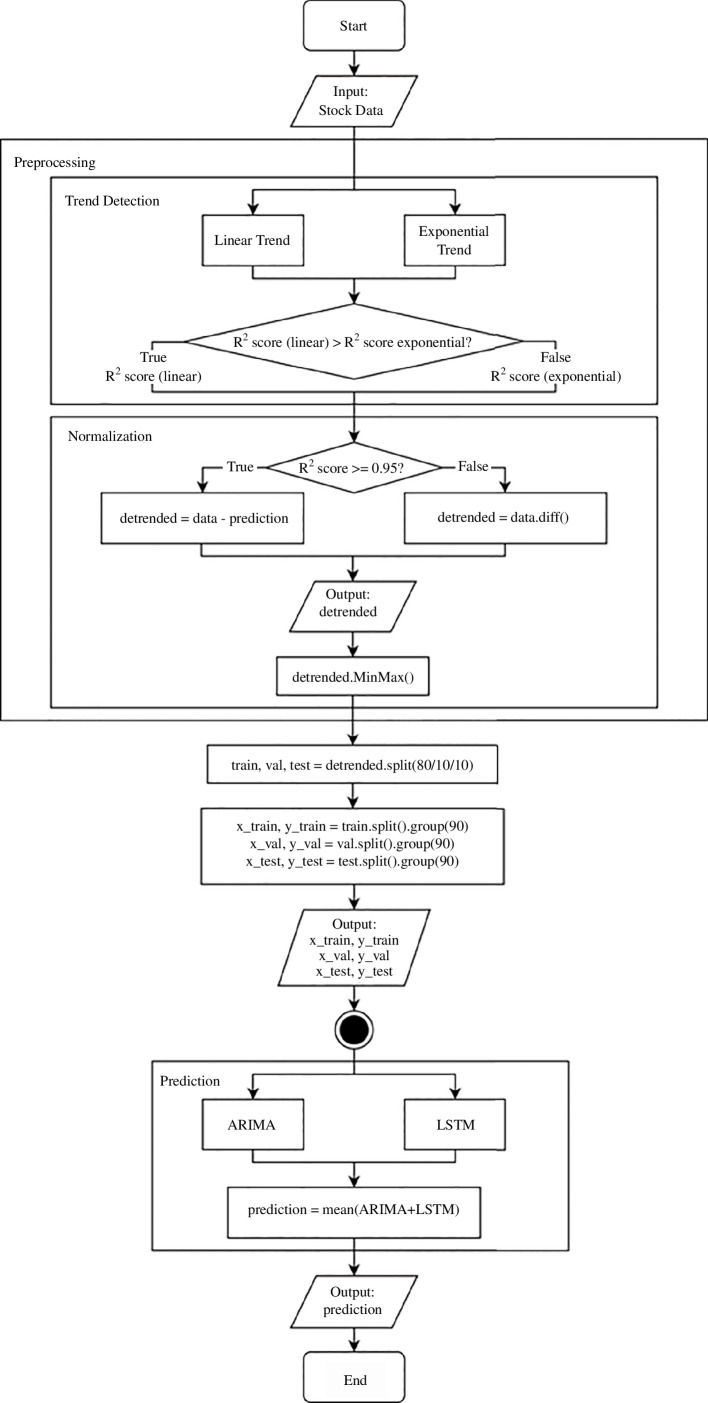
Flowchart of the proposed forecasting algorithm.

The purpose of the proposed approach is to reduce the impact of the shortcomings of each model on the overall forecast by balancing them with the other. For example, a recurrent neural network with a long short-term memory is less vulnerable to moving values in time series than an autoregressive moving average model, but can easily overlearn when there is insufficient data to make a prediction, which is not such a significant hindrance for a statistical model, as it makes predictions for the short term. The flowchart of the proposed algorithm is shown in figure 5.

Therefore, we chose LSTM and ARIMA models to create an ensemble method for forecasting financial markets, as they complement each other with their strengths: LSTM efficiently handles sequential data and captures long-term relationships, while ARIMA works well with time series that have linear relationships and seasonality. Other methods were considered but not included in the ensemble due to lower performance and difficulty in adapting to our forecasting task.

Another advantage of this approach is the possibility of parallelization. Because our method is set up like a bagging ensemble algorithm, both models used in it are largely independent of one another during their forecasting process. This means they can be easily split up between separate computational systems, which allows them to save time and still receive predictions of the same accuracy as if these models were running sequentially. This allows us to combine more algorithms in the same system: either completely new ones or different variations of those already present, for example with hyperparameters tuned for short-, mid- and long-time forecasting.

Additionally, due to the way the prediction is based on a certain preceding time period that shifts into the future rather than the overall data, we may parallelize the training process of the models by training them on these groups in parallel and later generalizing their results before validation and prediction. In such dynamic environments as financial markets, regular retraining and validation allow us to keep models up to date and ensure that forecast is based on patterns learned in conditions as close to the current ones as possible. In real-life applications, time is as important as accuracy because there is little use for forecasting what has already happened. Using parallel computing methods would greatly reduce forecasting time and give traders important information on which to base their market strategy as quickly and often as possible.

However, the latter approach may not suit the discussed LSTM and ARIMA models. The inherently sequential nature of these algorithms precludes parallelization within training examples, which becomes critical at longer sequence lengths, as memory constraints limit batching across examples. Thus splitting training data may be detrimental to the end result and therefore such implementation should be carefully considered and closely examined.

Another pitfall to consider is the computational capability available. When running models sequentially, we allocate all available power to one and then to another, and in the case of parallelization we split this capability between the algorithms in order to run them simultaneously. Without the aforementioned separate computational systems for these models, excess computational power that is underused during sequential application parallelization may harm the effectiveness of the approach.

## 3. Results

First of all, preliminary testing predicting the data of Nike shares (nke.us) was conducted with several intelligent methods. These were SVR, ARIMA, LSTM models and the ensemble algorithm of extreme gradient boosting XG-Boost. This allowed us to determine the best forecasting approaches for given data that will be chosen for use in future ensemble models. A summary of the performance of these algorithms is presented in table 2. As can be observed, the support vector regression algorithm with a sigmoid kernel function showed the worst result of all the studied methods, while ARIMA and LSTM showed the best results.

**Table 2.** Comparative table of forecasting metrics for different algorithms.

method name		RMSE	*R* ^2^
SVR	polynomial function	0.51699	−4.65866
	radial basis function	0.23987	−0.21814
	sigmoid function	68.06344	−98078.96983
ARIMA		0.62199	0.99013
LSTM		0.02544	0.98630
XG-Boost		0.12867	0.64946

RMSE, root mean square error.

It is to be noted that the other methods considered, namely different versions of support vector regression and the ensemble algorithm of extreme gradient boosting, are not the worst options for predicting financial markets because they performed worse in this study than recurrent networks with long short-term memory or the autoregressive moving average model. The problem lies in the data. These methods would probably perform much better with a different dataset. The problem with these methods is also the limited hyperparameters (compared with LSTM), the increased need for computing resources and time in the case of SVR, and additional manipulations of the dataset in the case of XG-Boost. These disadvantages can be avoided with additional optimization, cross-validation and other techniques, but the same applies to the methods that performed best in this study.

**Figure 6.**
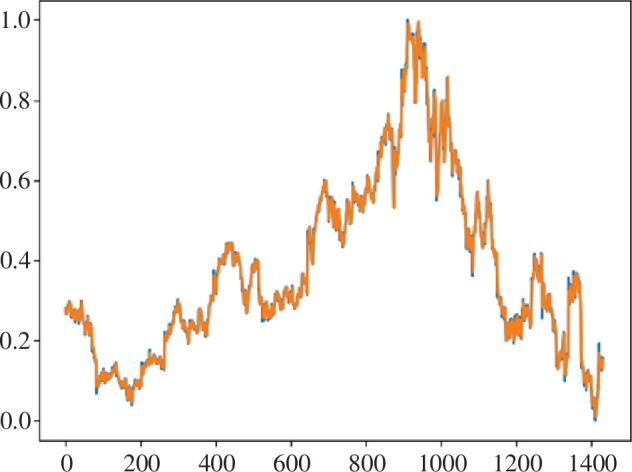
Prediction of test data using the proposed ensemble of methods (orange, actual data; blue, predicted data).

Now, let us consider the results of the implementation of the proposed ensemble for financial market prediction. Initially, the prediction was made on the previously considered and analysed dataset of Nike (nke.us) shares. In this case, the prediction graph is shown in figure 6.

**Figure 7.**
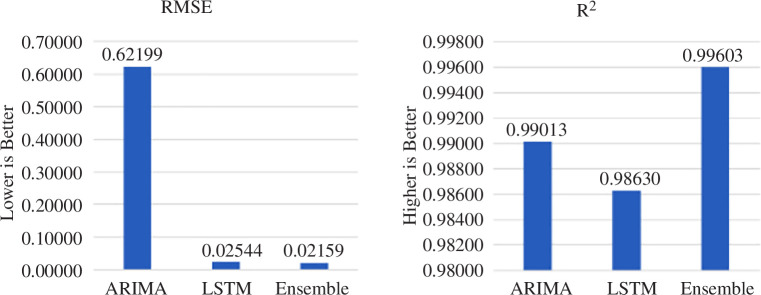
Comparison of RMSE and *R* ^2^ score metrics for ARIMA statistical model, LSTM neural network and proposed ensemble model.

As a result, the root mean square error (RMSE) of predicting the Nike (nke.us) stock dataset using our own model was 0.021588402062985842, which is a 15% improvement over the result obtained using LSTM, and the coefficient of determination of this model was 0.9960328101509566 (see figure 7).

**Figure 8.**
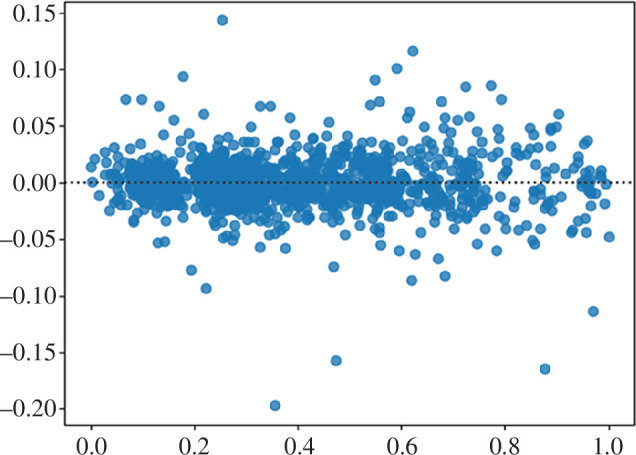
Residual plot for nke.us stock forecast by the proposed ensemble approach.

Additionally, as can be seen from the residual plot for the proposed ensemble model (see figure 8), such an approach fits this data quite well. Although there are some outlier values, most residuals spread out somewhat evenly (randomly) vertically on both sides of the horizontal regression line and there are no clearly identifiable trends visible on this plot, so we can conclude that the model indeed fits the data well.

Finally, to determine whether the observed differences are due to random chance, the Wilcoxon signed-rank statistical test was conducted [29]. In our case, the *p*-value for our data was 0.02288633, which is less than the threshold of 0.05. This means the sample is of the same distribution and the sample distributions are equal and such accuracy was not a coincidence.

## 4. Discussion

To further check the effectiveness of the proposed method, we will compare its efficiency with modern intelligent forecasting methods in financial markets.

**Figure 9.**
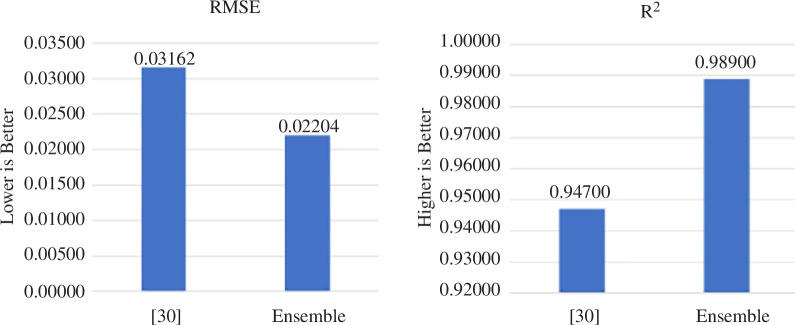
Comparison of RMSE and *R* ^2^ score metrics of nke.us stock for investigated method and proposed ensemble approach [30].

The first such comparison will be with [30]. In this article, the authors propose a complex recurrent neural model consisting of four LSTMs and four dropout layers, which together provide 260 065 training parameters. These data are divided into training and testing data in the proportion of 80/20, and the model is trained for 12, 25, 50 and 100 epochs. According to the authors of this research article, after 50 epochs of training, their prediction method showed a RMSE value of 0.03162 and a coefficient of determination of 0.947, which suggests that the model can track the evolution of changes in the data of Nike (nke.us). In our case, the model after training on the same data showed a mean square error of 0.022039, which is a 30.3% better prediction result compared with [30] (see figure 9).

Let us move on to another research article—this time, the authors explore the use of transformers to build financial market forecasts [31]. The authors point out that one of the key elements of transformers is the attention mechanism, which allows you to focus on specific information. In this study, a sparse attention transformer was used to forecast the closed share prices of Facebook (fb.us), as such a model requires less memory to efficiently process a large amount of historical data for more accurate forecasting. Thanks to this approach, the authors achieved a RMSE of 1.985 and a coefficient of determination of 0.892, which is not a very good result.

As for our proposed approach, our model was able to predict changes in the financial market (for the same fb.us dataset) with a RMSE of 0.04588, which is a 97.69% better result. The proposed method also outperformed the investigated approach with a 0.924824 *R* ^2^ score.

**Figure 10.**
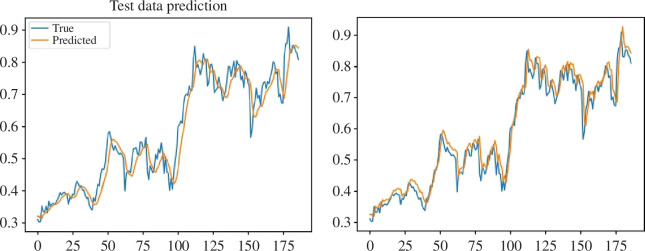
Prediction of fb.us using solely LSTM (left) and after generalizing forecasts of LSTM and ARIMA ensemble model (right).

Figure 10 shows the advantage of the chosen approach: in the conditions of a small amount of data, the recurrent neural network could not make an accurate forecast, because it was undertrained, so there was a shift in the prediction to the right relative to the actual data. However, the autoregressive model with a moving average was able to improve the forecasting accuracy at the moment of generalization of the forecast results. Thus, although the shift remained, it was significantly reduced.

**Figure 11.**
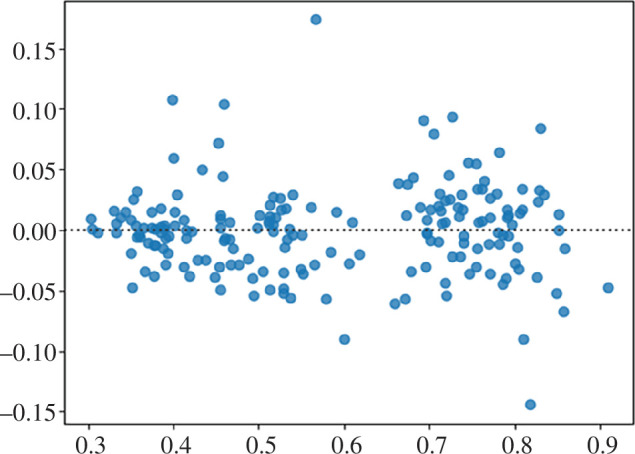
Residual plot for fb.us forecast stock by the proposed ensemble approach.

There are no identifiable patterns for the residual values on the residual plot (figure 11), as most residuals spread out somewhat evenly (randomly) vertically on both sides of the horizontal regression line and therefore this model indeed fits the data well.

**Figure 12.**
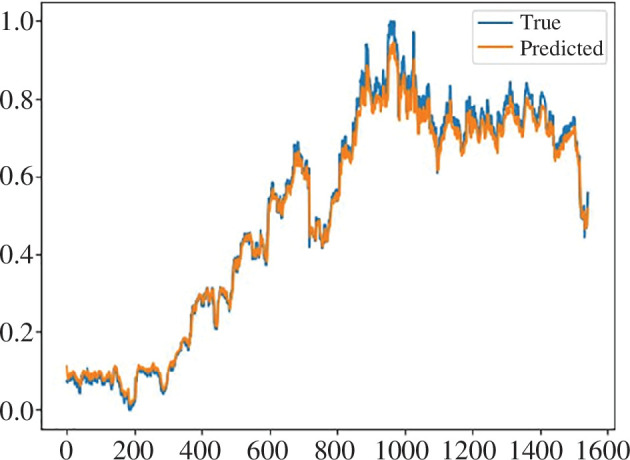
Results of predicting the wb.us test data using the proposed ensemble of LSTM and ARIMA models.

Now, let us take a look at another research paper on financial market forecasting, this time using an optimized deep recurrent neural network with long short-term memory [32]. Here, the authors examine the effectiveness of using the metaheuristic artificial rabbit optimization (ARO) algorithm to select hyperparameters for the LSTM model. With this approach, the authors hope to simplify the process of selecting the necessary hyperparameters to achieve the highest possible prediction accuracy when dealing with volatile data, such as the financial market. The optimization algorithm makes adjustments to the model’s hyperparameters as it passes through the training data. As a result of this approach, the authors obtained a RMSE of 0.074834 and a coefficient of determination of 0.925 for the resulting prediction. The authors compare these results with other LSTM models, conclude that their approach is the best of the considered ones, and suggest ways to further improve the method. Prediction results for the Walgreens Boots Alliance (wb.us) stock dataset using our proposed ensemble method are shown in figure 12.

**Figure 13.**
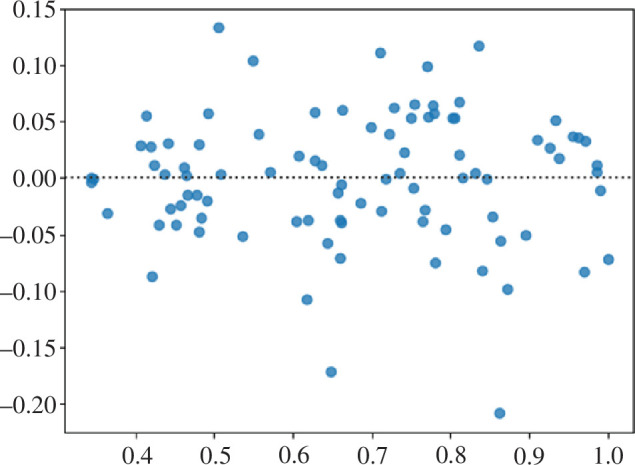
Residual plot for wb.us forecast stock by the proposed ensemble approach.

Once again, the residual values spread randomly on the residual plot (figure 13), as most residuals spread out somewhat evenly (randomly) vertically on both sides of the horizontal regression line and therefore this model indeed fits the data well.

Finally, the Wilcoxon signed-rank statistical test returned a *p*-value of 0.023732, which is less than the threshold of 0.05, therefore such accuracy was achieved because the sample is of the same distribution, and the sample distributions are equal.

In a scientific paper [8], the authors propose to improve the accuracy of stock market prediction with an expanded SentiWordNet (SWN) model. The goal is to train an extreme learning machine (ELM) and recurrent neural network (RNN) for stock price prediction by assigning sentiment scores to newly introduced stock market-related terms and applying the information gain method, resulting in the development of a new sentiment lexicon SSWN. The result is high forecasting accuracy for a number of stocks. For example, NVIDA (nvda.ua) stock was predicted with an accuracy of 87.35%

**Figure 14.**
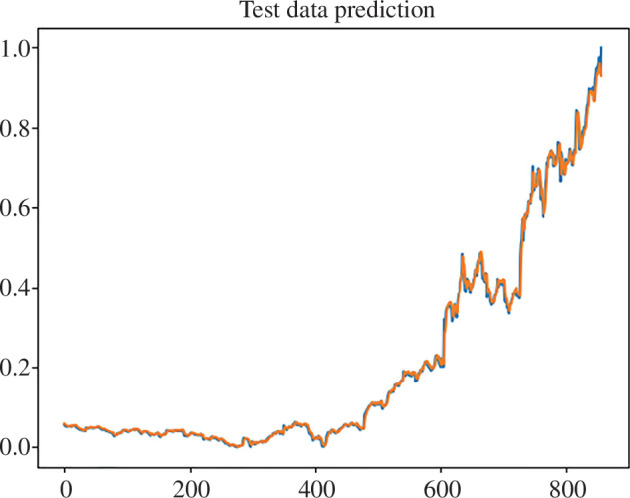
Results of predicting the nvda.us test data using the proposed ensemble of LSTM and ARIMA models.

As for our proposed approach, our model was able to predict changes for the same nvda.us dataset with a RMSE of 0.01312061 and an *R* ^2^ score of 0.99739 (figure 14).

**Figure 15.**
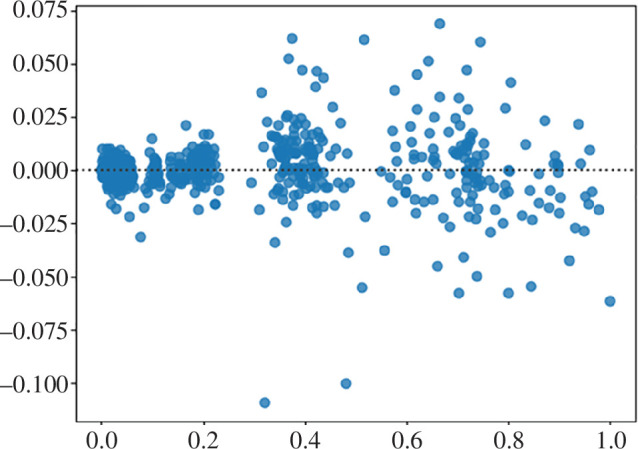
Residual plot for fb.us forecast stock by the proposed ensemble approach.

There are no identifiable patterns for the residual values on the residual plot (figure 15), as most residuals spread out somewhat evenly on the *y*-axis on both sides of the horizontal regression line and therefore this model indeed fits the data well.

All the obtained values of the roots of the mean square prediction error and the coefficients of determination for the above comparisons are presented in the corresponding tables 3 and 4.

**Table 3.** Comparison of the obtained RMSE of the considered methods and the proposed ensemble method.

dataset	RMSE for the considered methods	RMSE for the proposed ensemble method
nke.us	0.03162	0.02203
fb.us	1.95121	0.03797
wba.us	0.07483	0.02483

**Table 4.** Comparison of the determination coefficient of the considered methods and the proposed ensemble.

Dataset	*R* ^2^ for the considered methods	*R* ^2^ for the proposed ensemble method
nke.us	0.947	0.989
fb.us	0.892	0.948
wba.us	0.925	0.992

From the results obtained above, we can conclude that the proposed ensemble of methods is effective and can be used for different datasets, which in turn indicates the scalability of the proposed approach.

The paper does not address the potential problems with generalizing models to new market conditions that may change rapidly. The main challenges are the following:

— the lack of adaptability of models to sudden changes in the market, which can lead to a decrease in forecast accuracy;
— the possibility of retraining models on historical data that does not take into account new trends and anomalies;
— the difficulty of integrating new data in real time to keep forecasts up to date; and
— the risk is that models may not consider the interrelationships between different changing market factors.

These challenges require further research and developing methods that can adapt to dynamic market conditions.

## 5. Conclusion

In this paper, we develop a new ensemble approach to forecasting in financial markets. At the time of forecasting, we do not know the state of the financial market and whether LSTM or ARIMA made a better prediction (it depends on how well the data for forecasting is suitable for one or the other model), so we generalize to find a compromise that at best is a slight deviation from the best prediction, and at worst corrects the predicted value to the true value, thus reducing the final error.

The simulation results and three real datasets based on the RMSE criterion show that our proposed method has better performance than the LSTM recurrent neural network, the use of transformers and the optimized deep recurrent neural network with long short-term memory for building financial market forecasts, reducing the error by about 30.3%, 98.08% and 66.80%, respectively.

As we noted earlier, when forecasting financial markets, it is also necessary to take into account the speed of forecasting, so future research in this area should focus on parallelizing the ensemble forecasting procedure to minimize the decision-making time. To do this, it is worth focusing on the selection of modern technologies of distributed and parallel computing, which provide a significant advantage in the case of limited computing resources [33,34].

## Data Availability

Publicly available datasets were analysed in this study. This data can be found here [23].
